# Retrospective analysis of a VACM (vacuum-assisted closure and mesh-mediated fascial traction) treatment manual for temporary abdominal wall closure – results of 58 consecutive patients

**DOI:** 10.3205/iprs000098

**Published:** 2016-07-28

**Authors:** Christian Beltzer, Alexander Eisenächer, Steffen Badendieck, Dietrich Doll, Markus Küper, Stefan Lenz, Björn Dirk Krapohl

**Affiliations:** 1 Bundeswehr Hospital Ulm, Department of General, Visceral and Thorax Surgery, Ulm, Germany; 2 Bundeswehr Hospital Berlin, Department of General, Visceral and Thorax Surgery, Berlin, Germany; 3 St. Marienhospital, Vechta, Department of Colorectal Surgery, Vechta, Germany; 4 Klinikum Nauen, Department of General and Visceral Surgery, Nauen, Germany; 5 St. Marien-Krankenhaus, Berlin, Department of Plastic and Hand Surgery, Berlin, Germany

**Keywords:** temporary abdominal closure, mesh-mediated fascial traction, fascial closure rate, open abdomen, peritonitis, vacuum-assisted closure, laparostoma

## Abstract

**Introduction: **The optimal treatment concept for temporary abdominal closure (TAC) in critically ill visceral surgery patients with open abdomen (OA) continues to be unclear. The VACM (vacuum-assisted closure and mesh-mediated fascial traction) therapy seems to permit higher delayed primary fascial closure rates (FCR) than other TAC procedures.

**Material and methods: **Patients of our clinic (n=58) who were treated by application of a VAC/VACM treatment manual in the period from 2005 to 2008 were retrospectively analysed.

**Results: **The overall FCR of all patients was 48.3% (95% confidence interval: 34.95–61.78). An FCR of 61.3% was achieved in patients who had a vicryl mesh implanted at the fascial level (VACM therapy) in the course of treatment. Mortality among patients treated with VACM therapy was 45.2% (95% CI: 27.32–63.97).

**Conclusions:** The results of our own study confirm the results of previous studies which showed an acceptable FCR among non-trauma patients who were treated with VACM therapy. VACM therapy currently appears to be the treatment regime of choice for patients with OA requiring TAC.

## Zusammenfassung

**Einleitung: **Das optimale Behandlungskonzept eines temporären Bauchdeckenverschlusses (temporary abdominal closure, TAC) bei kritisch kranken viszeralchirurgischen Patienten mit offenem Abdomen („open abdomen“, OA) ist weiterhin unklar. Durch eine VACM-Therapie (vacuum-assisted closure and mesh-mediated fascial traction) scheinen sich gegenüber anderen Verfahren des TAC höhere Faszienverschlussraten (delayed primary fascial closure rate, FCR) realisieren zu lassen.

**Material und Methoden: **Patienten unserer Klinik (n=58), welche in den Jahren 2005 bis 2008 mittels eines VAC/VACM-Behandlungsmanuals behandelt wurden, wurden retrospektiv analysiert.

**Ergebnisse: **Die FCR aller Patienten betrug insgesamt 48,3% (95%-Konfidenzintervall: 34,95–61,78). Bei Patienten, bei denen im Verlauf ein Vicryl-Netz auf Faszienebene implantiert wurde (VACM-Therapie), konnte eine FCR von 61,3% realisiert werden. Die Letalität der mittels VACM therapierten Patienten betrug 45,2% (95%-KI: 27,32–63,97).

**Schlussfolgerung: **Die Ergebnisse der eigenen Untersuchung bestätigen bisherige Studienergebnisse, die eine akzeptable FCR bei non-trauma-Patienten durch Anwendung der VACM-Therapie zeigen konnten. Die VACM-Therapie scheint aktuell Therapiekonzept der ersten Wahl bei Patienten mit OA und Indikation zum TAC zu sein.

## Introduction

Severe intraabdominal infections with peritonitis, bowel obstruction, pancreatitis, abdominal compartment syndrome and planned second-look surgery are indications for open abdomen (OA) management with temporary abdominal closure (TAC). Various techniques can be applied for TAC management of critically ill visceral surgery patients [1], [2], [3]. Meanwhile, in particular treatment concepts that are based on the principle of negative pressure generation (negative pressure wound therapy, NPWT) have become established, such as VAC or VACM therapy [4]. NPWT can be used to drain off intraabdominal secretions and it has a positive effect on the treatment of abdominal compartment syndrome [5]. It seems that high FCRs can be achieved by application of VACM therapy, which is based on the principle of mesh-mediated fascial traction [6], [7]. Patients with TAC may develop enteroatmospheric fistulas (EAF), which constitute a complication that is difficult to treat. Reported fistula rates during application of NPWT are 21% [8]. However, a causal connection between the occurrence of EAF and NPWT in open abdomen is questionable [9], [10]. Due to insufficient evidence, the optimal therapy for open abdomen continues to be unclear [11], [12]. In Germany, a multicentric laparostoma register of the German Society for General and Visceral Surgery (DGAV) has been established as a contribution to improving the data available on treatment of the open abdomen in order to be able to derive evidence-based therapy recommendations from these data in future [13]. As a result, the major objectives in the treatment of patients with OA are as follows: achieving fascial closure in the course of treatment and avoiding the occurrence of EAF [14]. Various studies showed that VACM therapy was associated with higher FCRs and lower incidence of EAF during treatment than VAC therapy alone [15], [16], [17]. In this retrospective study, the FCR, the mortality rate and the incidence of enteroatmospheric fistulas among patients of our department who were treated using a combined VAC/VACM treatment manual were to be retrospectively analysed.

## Material and methods

### Data collection and statistical analysis

This study retrospectively covers all patients of our department who underwent open abdomen management with TAC for conditions of non-traumatic origin in the period from 2005 to 2008 (n=58). The following patient characteristics and therapy results were analysed: age, sex, indication for open abdomen treatment, severity of disease at the beginning of treatment with TAC, using the Simplified Acute Physiology Score (SAPS II), SAPS II for survivors and non-survivors, length of stay (LOS), FCR, mortality rate and EAF rate during treatment.

After establishing a TAC in the intensive care unit, the SAPS-II was determined as described by Le Gall et al. [18].

The mean value, standard deviation, median and 95% confidence interval were considered in the data given. The analysis was conducted using SPSS Version 16.0. The level of significance was set at p≤0.05. 

### Treatment manual

**Figure 1:**
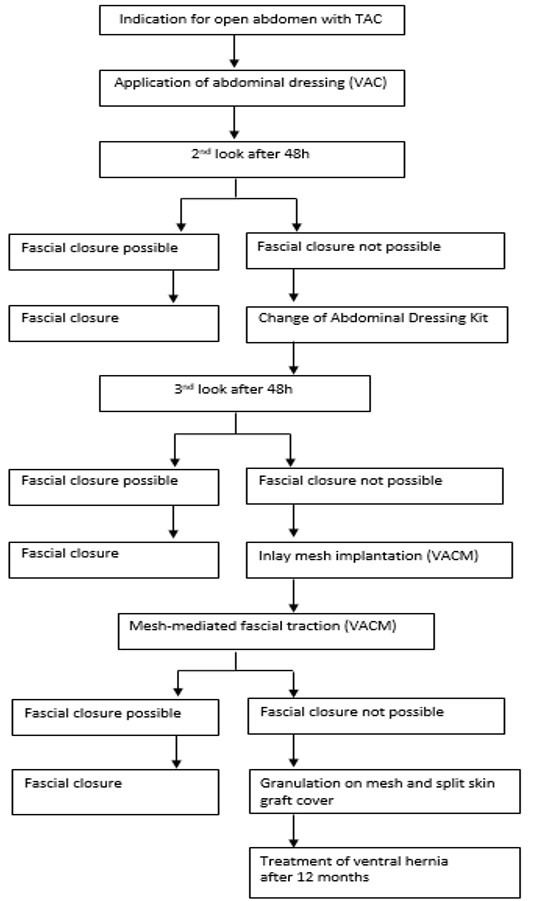
Treatment manual for patients requiring open abdomen (OA) management

All patients were treated using a combined VAC/VACM therapy in accordance with a specified treatment manual (Figure 1 (Fig. 1)). Since there was an indication for TAC, a primary commercial V.A.C.^®^ Abdominal-Dressing^TM^ (Kinetic Concepts Inc., USA) was applied. Planned surgical revisions were subsequently performed every 48 hours. In patients with clean intraabdominal conditions, the fascia was closed during the first surgical revision (second-look surgery). Where fascial closure could not be accomplished at that time, the Abdominal-Dressin^TM^ was changed. Where fascial closure could still not be achieved during the second surgical revision after application of the VAC dressing, polyglactin mesh (Ethicon, Germany) was sutured as inlay between the fascial edges (VACM) to prevent further retraction of the fascial edges. During the subsequent surgical revisions, intraabdominal access was achieved by incision of the polyglactin mesh along the median line to leave the fascial edges intact. At the end of surgery, the polyglactin mesh was closed by an overlapping suture along the median line in order to approximate the fascial edges and thus to reduce the laparostoma. 

## Results

### Age and sex

39.7% of all patients were female and 60.3% were male. The average age of all patients was 67.4 ± 15.7 years (median: 68.5 years). With an average age of 74.6 ± 12.7 years (median: 80.0 years), the female patients were significantly older than the male patients with an average age of 62.7 ± 15.9 years (median: 65.0 years) (p=0.005). 

**Figure 2:**
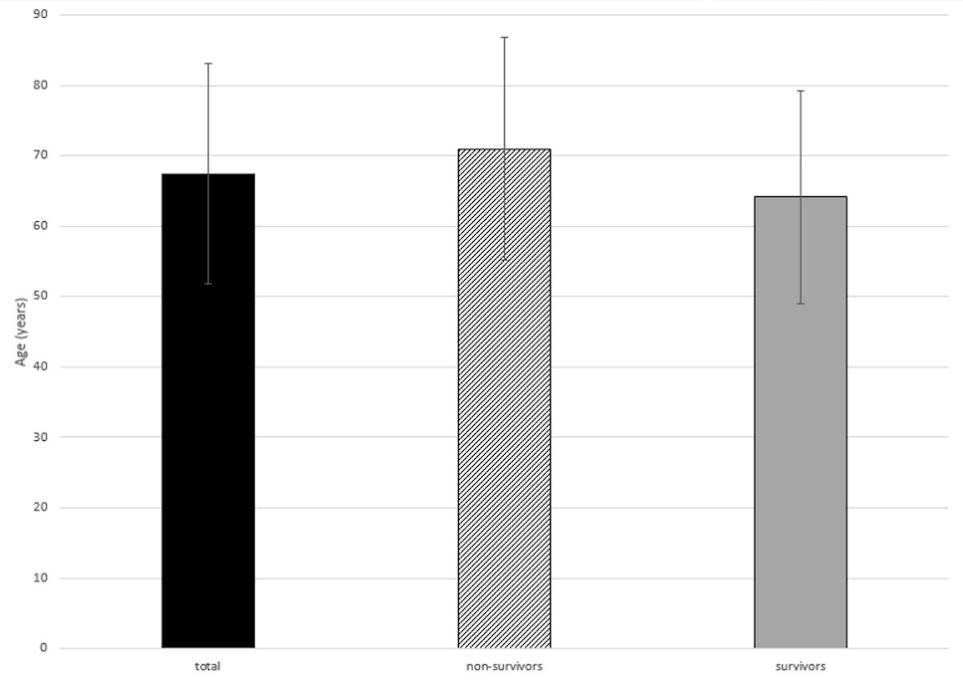
Age of own patient group: total, non-survivors, survivors

The average age of the patients who survived treatment was 64.2 ± 15.1 years (median: 67.0 years). The average age of the patients who died during treatment was 71.0 ± 15.8 years (median: 79.5 years) (Figure 2 (Fig. 2)).

### Indication for open abdomen management 

**Figure 3:**
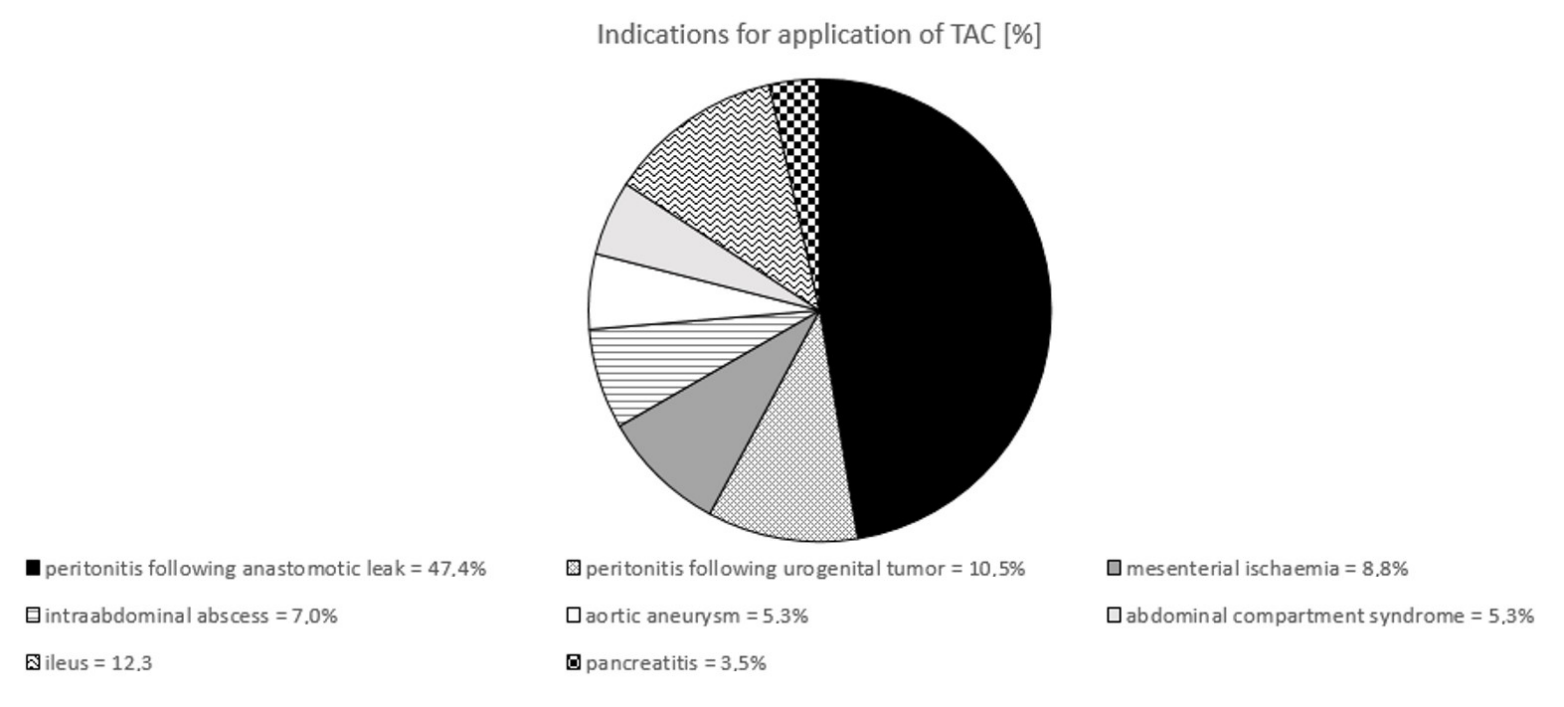
Indication for application of TAC in own patient group

The most common indication for open abdomen management was secondary peritonitis following visceral or urological surgery (57.9%). The second most common indication was bowel obstruction (12.3%), followed by mesenterial ischaemia (8.8%) and intraabdominal abscesses (7.0%). Other indications were abdominal compartment syndrome (5.3%), abdominal aortic aneurysm (5.3%) and pancreatitis (3.5%) (Figure 3 (Fig. 3)). 

### Simplified Acute Physiology Score (SAPS II)

**Figure 4:**
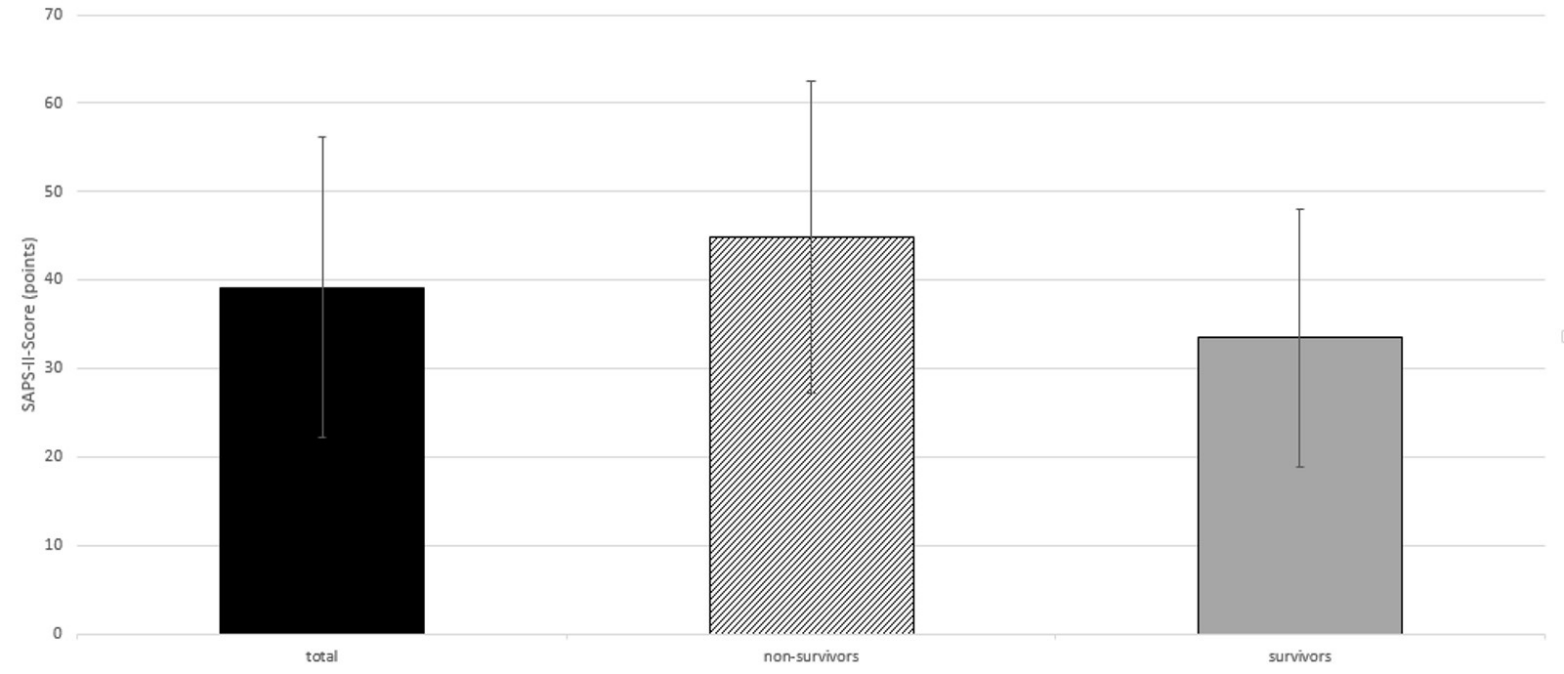
SAPS II of own patient group at the beginning of treatment: total, non-survivors, survivors

At the beginning of intensive care treatment, the mean SAPS II of all patients was 39.2 ± 17.0 points (median: 35.0 points). The SAPS II of the patients who died during treatment was 44.9 ± 17.6 points (median: 50.0 points). The patients who survived treatment had a significantly lower SAPS II of 33.5 ± 14.5 points (median: 31.0 points) (p=0.027) (Figure 4 (Fig. 4)).

### Delayed primary fascial closure rate (FCR)

**Figure 5:**
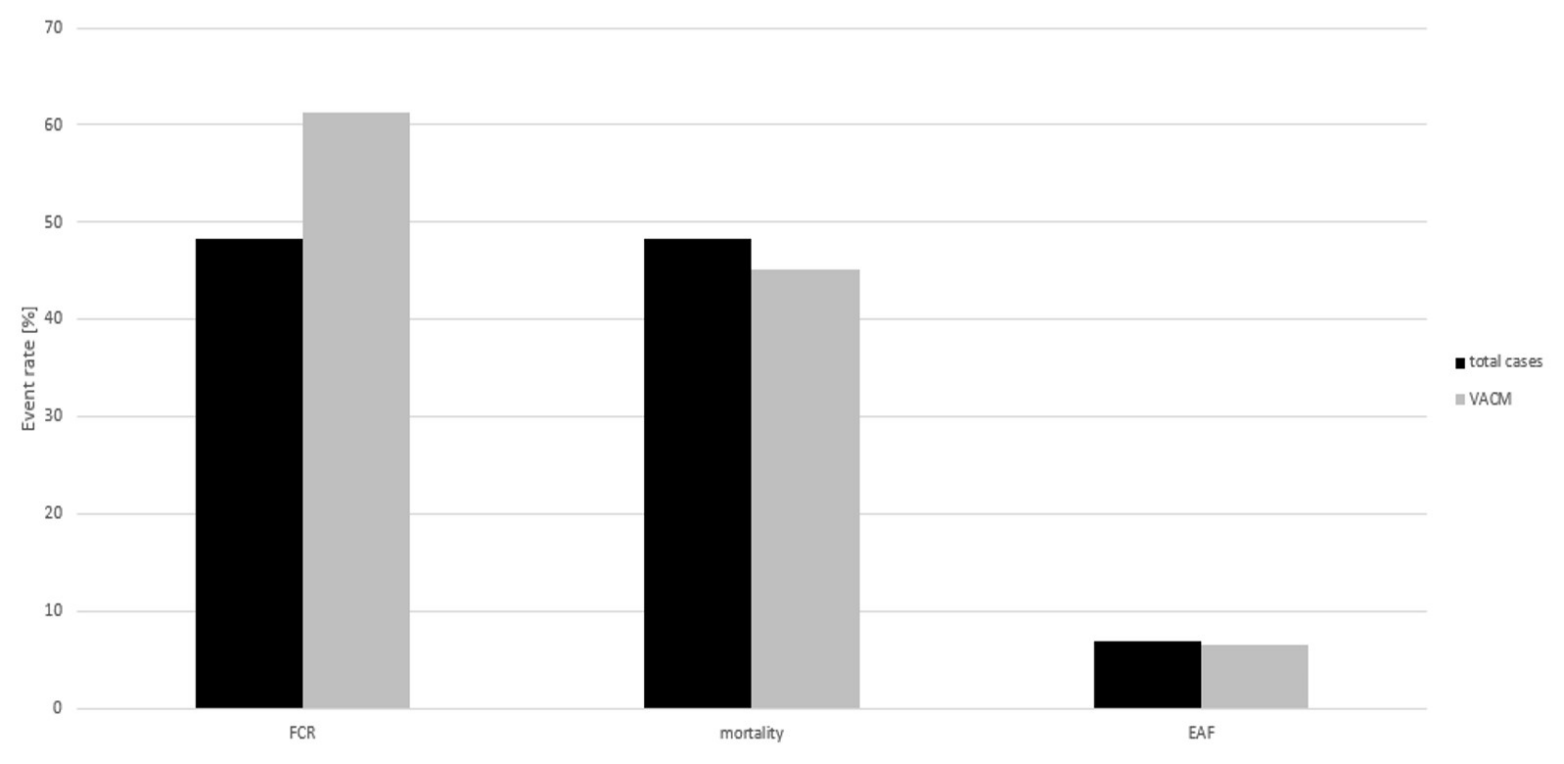
Event rates of FCR, mortality and EAF, total cases and VACM therapy

The FCR of all patients was 48.3% (28/58, 95% CI: 34.95–61.78), the FCR of patients who were treated with VACM was 61.3% (19/31, 95% CI: 42.19–78.15) (Figure 5 (Fig. 5)). 

### Mortality

The overall mortality rate was 48.3% (28/58, 95% CI: 34.95–61.78), and among patients who were treated with VACM the mortality rate was 45.2% (14/31, 95% CI: 27.32–63.97) (Figure 5 (Fig. 5)). 

### Enteroatmospheric fistulas (EAF)

Enteroatmospheric fistulas occurred in 6.9% (4/58, 95% CI: 1.91–16.73) of the patients during the course of treatment. The rate of fistula formation among patients treated with VACM therapy was 6.5% (2/31, 95% CI: 0.42–11.91) (Figure 5 (Fig. 5)).

### Length of stay (LOS)

**Figure 6:**
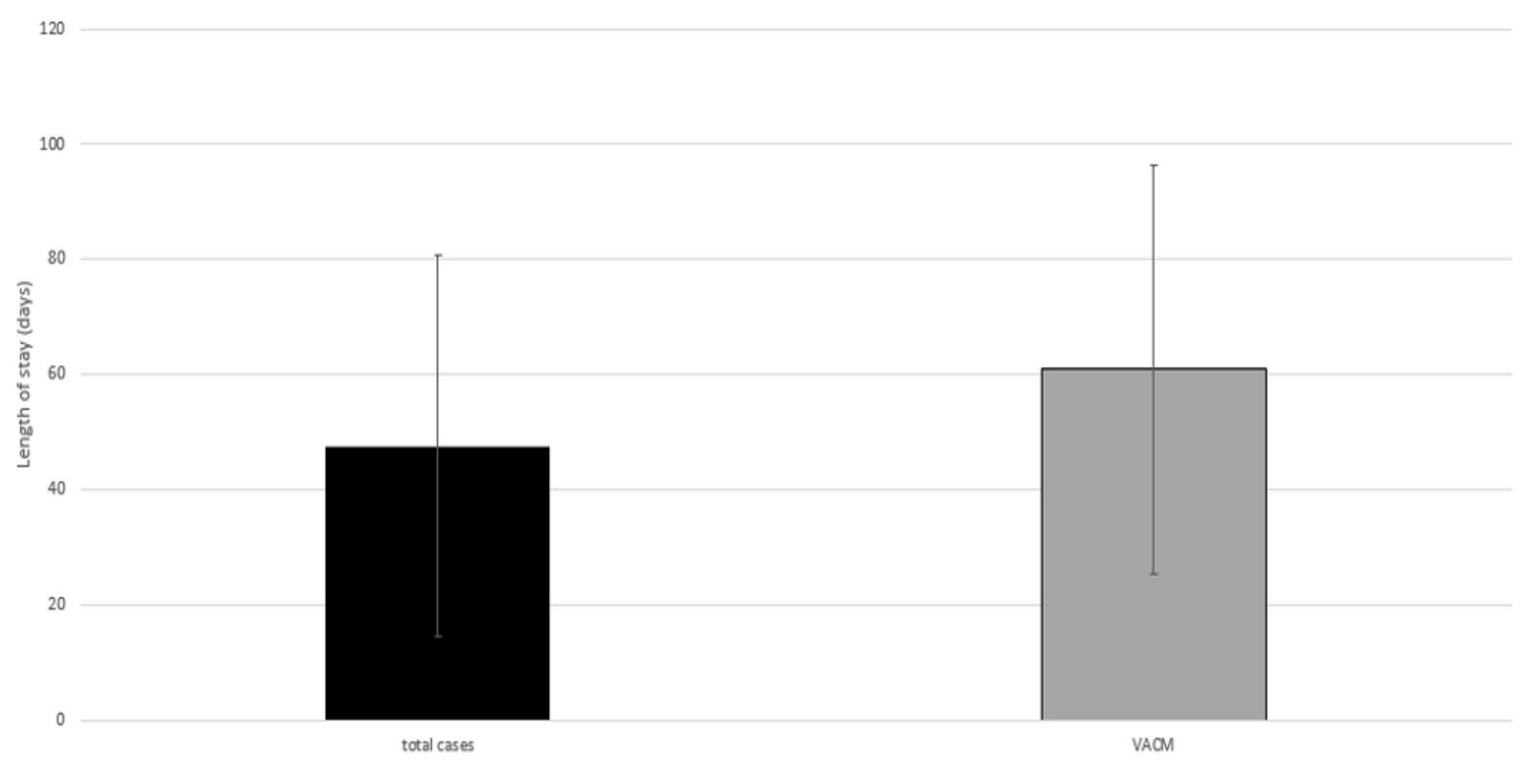
Length of hospital stay (LOS): total cases and VACM therapy

The average duration of inpatient treatment of all patients was 47.6 days (median: 45.0; 95% CI: 38.32–56.79) (Figure 6 (Fig. 6)). The mean length of hospital stay of patients who were successfully treated by placement of polyglactin mesh (VACM) was 80.1 days (median: 72.0; 95% CI: 64.38–95.86), whereas the mean length of stay of patients who achieved early fascial closure was 42.0 days (median: 36.0; 95% CI: 28.66–55.34). The LOS can be significantly reduced by early abdominal closure if no relevant complications occur during the course of treatment (p=0.002) (Figure 6 (Fig. 6)).

## Discussion

The patients who underwent VACM therapy in our department had a high mortality rate of 45.2%. However, the relevant literature also indicates mortality rates >30% for patients with open abdomen and TAC [2], in individual cases mortality rates >50% have been described [19]. Mortality is particularly high in patient groups with secondary peritonitis: in a prospective study by Fortelny et al. [20] which only included patients with a septic intraabdominal focus it was even 55.2%. 

Since patient age is an important determinant of the outcome, it must also be considered when interpreting the results of treatment: Vogel et al. [21] found that advanced age was associated with significantly higher complication rates and prolonged treatment courses (p<0.001). In 2011, Acosta et al. [22] reported a significant influence of advanced age on mortality in patients with temporary abdominal wall closure (p=0.027).

The FCR reported after VACM therapy was 61.25% and thus higher than in studies where VAC therapy was applied alone (33%) [23], but it was lower than in other studies which reported FCRs of 76% [22], 78% [7] and 87% for patients who underwent VACM therapy [16]. In the publication by Willms et al. [16], the average age of the patients was 53 years compared to an average age of 68.5 years of our own patient group. 

In some cases, the indications for using TAC differ considerably between our own patient group and the populations described in the literature (trauma vs. non-trauma patients). 

It is known that only relatively low FCRs can be achieved among patients with secondary peritonitis compared to patients without septic intraabdominal foci [15], [24]. Extraabdominal infections in patients who underwent TAC were also associated with a lower FCR [21]. Patients with severe peritonitis often require lengthy treatment, because early fascial closure is usually not possible in such cases. At the same time, it was reported that the chances of fascial closure decrease with increasing duration of treatment [3]. 

The EAF rate of 6.5% during VACM therapy corresponds to the data described in the literature, although individual authors published fistula rates of 0% for patients who underwent VACM therapy [16]. 

The severity of disease at the beginning of treatment (SAPS II) is an important prognostic factor. The findings in our patient group show that a high SAPS II is associated with increased mortality. Such a correlation is obvious, but a comparison with the literature is difficult: in some studies intensive medical scores are not indicated regularly and sometimes other score systems (APACHE II, ISS) are used [2]. 

The length of hospital stay (LOS) is significantly prolonged after mesh implantation. The LOS can be significantly reduced by rapid abdominal closure if no relevant complications occur during the course of treatment (p=0.002). 

The treatment manual used was already described in a similar form in other publications, and even higher FCRs were reported in some of these studies [16], [25]. 

There are no valid data on the optimum time for performing additional mesh implantation and starting VACM therapy. It is unclear whether even higher FCRs can be achieved in the course of treatment by implanting mesh already during initial establishment of TAC. 

The validity of our own study is limited by the fact that the patients were not classified according to their respective intraabdominal findings on the basis of uniform standards, as suggested by Björck et al. [26]. Therefore, as in many other studies, the described results cannot easily be transferred to other patient populations. 

The analysed patients were treated in the period from 2005 to 2008. The materials (Abdominal Dressing^TM^, KCI, poylglactin mesh) and therapy concepts (VACM) used for these patients are still commonly used. The data presented here can therefore be used to extend current evidence, although new commercial systems, such as ABThera^TM^ (Kinetic Concepts Inc., USA) are meanwhile available for TAC therapy and the first treatment outcomes have been published [27]. 

Furthermore, the retrospective evaluation of data, the lack of a control group and the single-centre character of the study are limiting factors in the interpretation of results. 

## Conclusions

VACM therapy should be used whenever possible, especially for non-trauma patients requiring TAC. By using the principle of mesh-mediated fascial traction, it is possible to achieve acceptable fascial closure rates and low rates of enteroatmospheric fistulas during the course of treatment. In future it may be possible to derive evidence-based recommendations for treatment from the laparostoma register of the German Society for General and Visceral Surgery (DGAV).

## Notes

### Competing interests

The authors declare that they have no competing interests.
